# Supplementary material for: Fingerprinting of Volatile Organic Compounds for the Geographical Discrimination of Rice Samples from Northeast China
Source: Foods. 2022 Jun 9;11(12):1695. doi: 10.3390/foods11121695 (PMC9222519; doi:10.3390/foods11121695)
Supplement: Supplementary file 1 [file foods-11-01695-s001.zip › foods-1715438-supplementary.pdf]

SUPPLEMENTARY INFORMATION

(Table S1- Table S2; Figure S1-Figure S2)

Metabolomic fingerprinting of volatile organic compounds for the geographical discrimination of rice samples from Northeast China

Sailimuhan Asimi [12#], Ren Xin [12#], Zhang Min[12]*, Li Sixuan[12], Guan Lina[12], Wang Zhenhua [12], Shan Liang[12], Wang Ziyuan [12]

1. Beijing Advanced Innovation Center for Food Nutrition and Human Health, Beijing Technology and Business University, Beijing 100048, China;

2. Beijing Engineering and Technology Research Center of Food Additives, Beijing Technology and Business University, Beijing 100048, China

Address: Beijing Technology & Business University, 11 Fucheng Road, Beijing, China, 100048.

Tel : 86-10- 68984547

[#]These authors contributed equally to this work and should be considered co-first authors

Correspondence:

Zhang Min (zmin@th.btbu.edu.cn)

Table S1 Geographical origin of the rice samples

**Samples**	**Variety**	**Region**	**Prefecture**
1	Longjing 31	Heilongjiang	Qiqihar
2	Longjing 39	Heilongjiang	Qiqihar
3	Longjing 46	Heilongjiang	Qiqihar
4	Longjing 71	Heilongjiang	Qiqihar
5	Suijing 306	Heilongjiang	Suihua
6	Longke 3	Heilongjiang	Qiqihar
7	Jinyu 1	Heilongjiang	Qiqihar
8	Suijing 18	Heilongjiang	Suihua
9	Suijing 28	Heilongjiang	Suihua
10	Suijing 302	Heilongjiang	Suihua
11	Jijing303	Jilin	Changchun
12	Jijing525	Jilin	Changchun
13	Jijing816	Jilin	Changchun
14	Jijing 830	Jilin	Tonghua
15	Jiyang 1	Jilin	Tonghua
6	Jiyang 100	Jilin	Tonghua
17	Tonghe 877	Jilin	Tonghua
18	Tonghe 822	Jilin	Tonghua
19	Tongyuan 568	Jilin	Tonghua
20	Tongke 29	Jilin	Tonghua
21	Liaojing 212	Liaoning	Shenyang
22	Liaojing 436	Liaoning	Shenyang
23	Liaojing 433	Liaoning	Shenyang
24	Liaojing 419	Liaoning	Shenyang
25	Liaoxing 21	Liaoning	Shenyang
26	Yanjing 927	Liaoning	Panjin
27	Yanjing 337	Liaoning	Panjin
28	Yanfeng 47	Liaoning	Panjin
29	Yanjing 939	Liaoning	Panjin
30	Yanjing 219	Liaoning	Panjin

Table S2 VOCs identified in the rice sample with GC–MS. (μg/kg)

No	Compound	CAS	Molecular Formula	RI	Heilongjiang	Jilin	Liaoning
1	Decane	124-18-5	$C_{10}H_{22}$	1000	0.58−3.24	0.35−5.42	0.28−0.62
2	Undecane	1120-21-4	$C_{11}H_{24}$	1100	1.78−6.59	0.71−12.97	2.29−3.43
3	2-methyl-decane	6975-98-0	$C_{11}H_{24}$	1064	0.46−7.14	2.1−32.98	15.02−15.21
4	Dodecane	112-40-3	$C_{12}H_{26}$	1200	8.87−22.41	4.72−28.37	17.45−59.48
5	Tridecane	629-50-5	$C_{13}H_{28}$	1300	3.15−27.48	2.98−26.78	56.34−64.59
6	Tetradecane	629-59-4	$C_{14}H_{30}$	1400	28.91−119.44	49.2−115.99	153.71−246.26
7	Hexadecane	544-76-3	$C_{16}H_{34}$	1600	2.44−9.72	2.28−19.27	12.26−16.56
8	2,6,10,14-tetramethyl-hexadecane	638-36-8	$C_{20}H_{42}$	1792	2.69−7.93	4.85−14.85	8.06−15.51
9	Styrene	100-42-5	C_8H_8	1254	3.27−13.22	2.13−15.29	23.17−45.29
10	2,6-dimethyl-decane	13150-81-7	$C_{12}H_{26}$	1112	1.15−7.54	2.49−10.35	6.43−23.0
11	2-methyl-dodecane	1560-97-0	$C_{13}H_{28}$	1264	6.89−129.06	9.33−33.9	158.78−204.51
12	2,6,10-trimethyl-dodecane	3891-98-3	$C_{15}H_{32}$	1366	8.72−138.3	8.13−43.86	54.8−286.06
13	Heptanal	111-71-7	$C_7H_{14}O$	1182	8.70−8.70	4.70−9.75	n.d.
14	(E,E)-2,4-nonadienal	5910-87-2	$C_9H_{14}O$	1216	9.71−71.91	0.58−12.93	28.31−61.67
15	Nonanal	124-19-6	$C_9H_{18}O$	1104	8.29−17.85	2.51−44.89	n.d.
16	(E)-2-heptenal	18829-55-5	$C_7H_{12}O$	1334	5.03−5.03	81.48−277.59	289.36−374.42
17	Decanal	112-31-2	$C_{10}H_{20}O$	1206	1.18−18.40	7.69−12.53	8.94−47.46
18	Pentadecanal	316249	$C_{15}H_{30}O$	1715	1.59−6.82	0.46−18.36	2.36−2.88
19	Hexadecanal	629-80-1	$C_{16}H_{32}O$	1817	0.52−2.10	1.44−2.84	0.81−0.88
20	(E) 6, I0-dimethyl-5,9 undecadien-2-one	3796-70-1	$C_{13}H_{22}O$	1876	4.79−15.45	12.62−29.42	11.50−19.01
21	6,10-dimethyl-2-undecanone	1604-34-8	$C_{13}H_{26}O$	1408	13.76−25.85	6.99−20.19	12.11−19.78
22	Hexanol	626-93-7	$C_6H_{14}O$	1211	3.90−24.12	1.71−27.2	19.03−51.06
23	1-octanol	111-87-5	$C_8H_{18}O$	1558	0.42−4.66	1.28−8.39	6.22−24.36
24	n-heptadecanol	1454-85-9	$C_{17}H_{36}O$	1984	0.27−0.85	1.26−3.46	1.49−1.69
25	2-hexyl-1-decanol	2425-77-6	$C_{16}H_{34}O$	1504	8.10−32.28	12.09−24.13	21.35−37.47
26	3,7,11-trimethyl-1-dodecanol	6750-34-1	$C_{15}H_{32}O$	1571	1.88 −5.75	1.13−7.99	12.54−49.56
27	Octanoic acid	124-07-2	$C_8H_{16}O_2$	2050	0.14 −0.40	0.27−5.78	0.26−0.31
28	n-Hexadecanoic acid	57-10-3	$C_{16}H_{32}O_2$	1968	1.05 −2.03	2.02−8.28	1.58−2.38
29	Hexanoic acid	142-62-1	$C_6H_{12}O_2$	1849	0.09 −0.09	n.d.	0.42−0.42
30	Nonanoic acid	112-05-0	$C_9H_{18}O_2$	2173	0.48 −1.52	0.85−2.92	0.95−8.01
31	Benzyl alcohol	100-51-6	C_7H_8O	1877	1.89 − 6.22	3.61−5.57	3.85−5.21
32	Toluene	108-88-3	C_7H_8	1036	0.88 −11.29	0.61−3.05	1.17−1.96
33	2-butyl-1-octanol	735273	$C_{12}H_{26}O$	1277	11.95−80.42	6.69−41.94	55.07−79.89

RI: retention index.

n.d.: not detected.

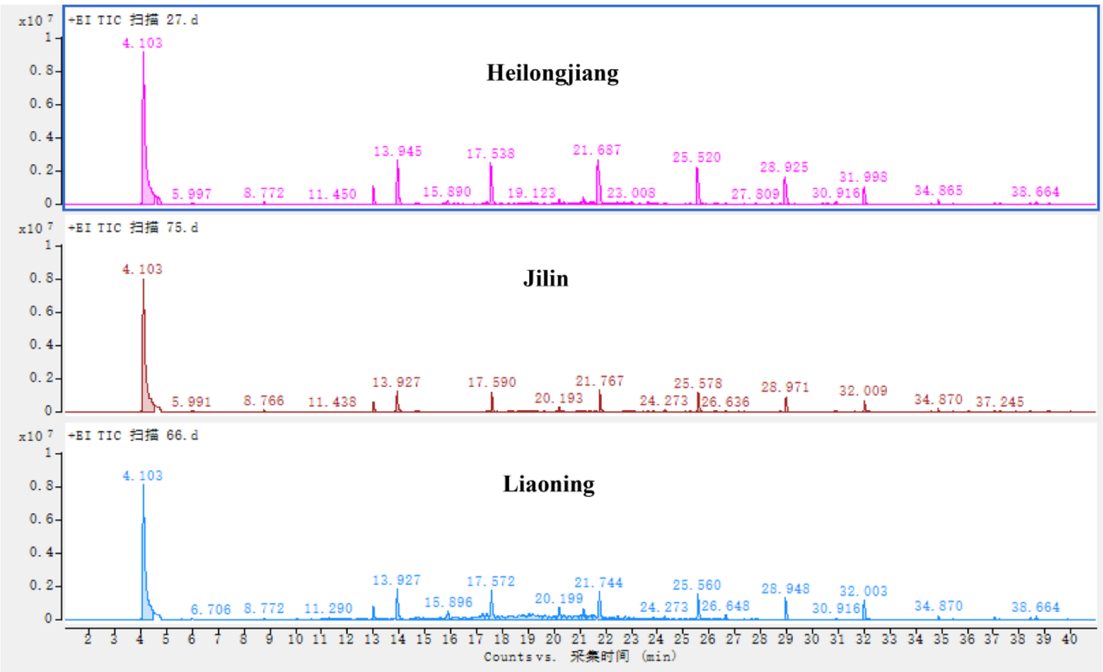

Figure S1. Total ion chromatogram of VOCs profile of Heilongjiang, Liaoning, and Jilin rice samples were obtained from HS-SPME-GC-MS.

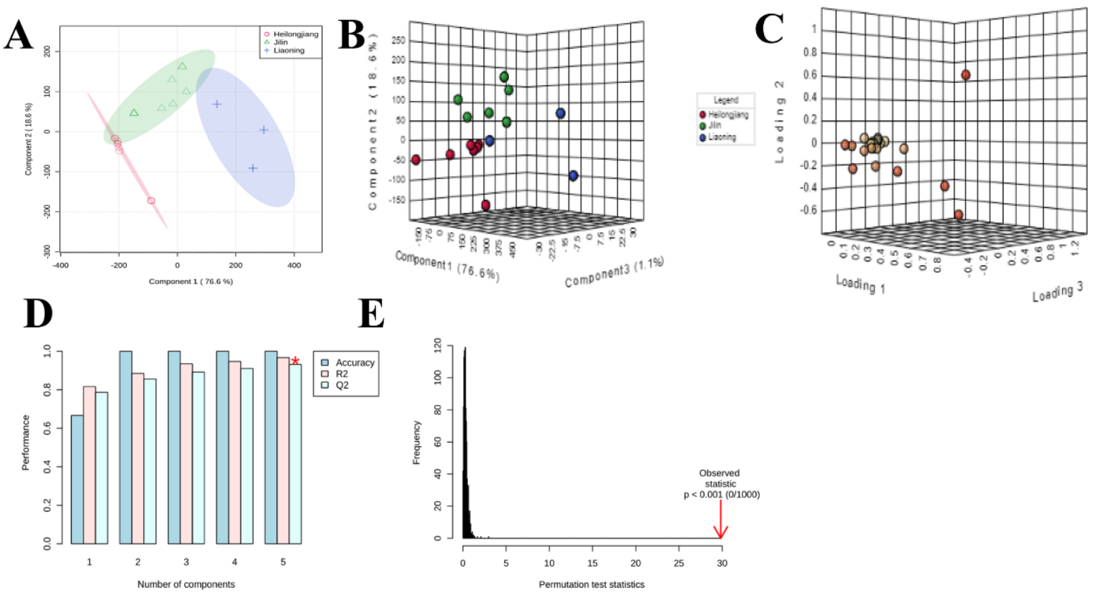

Figure S2. Significant differences in VOCs profile of Heilongjiang, Jilin, and Liaoning rice samples A) PLS-DA scores plot for the first component (76.6%) Vs second component (18.6%), indicating discrimination between Heilongjiang (red color), Jilin (green color), and Liaoning (blue color) rice samples. B) Three-dimensional PLS-DA scores plot. C) Three-dimensional PLS-DA loading plot. D) PLS-DA classification using the different number of components. Bar plots show the three performance measures (prediction accuracy, R2, and Q2) using different components. The red '*' indicates the best values of the currently selected measures (Q^2). E) PLS-DA model validation by permutation tests based on the separation distance. The p-value based on permutation is $P < 0.001$ (0/1000).
